# Motion correction for functional MRI with three‐dimensional hybrid radial‐Cartesian EPI

**DOI:** 10.1002/mrm.26390

**Published:** 2016-09-08

**Authors:** Nadine N. Graedel, Jennifer A. McNab, Mark Chiew, Karla L. Miller

**Affiliations:** ^1^ FMRIB Centre for Functional MRI of the Brain John Radcliffe Hospital, University of Oxford Oxford United Kingdom; ^2^ Department of Radiology Stanford University Stanford California USA

**Keywords:** functional MRI, 3D EPI, radial‐Cartesian EPI, motion correction, self‐navigated, golden angle

## Abstract

**Purpose:**

Subject motion is a major source of image degradation for functional MRI (fMRI), especially when using multishot sequences like three‐dimensional (3D EPI). We present a hybrid radial‐Cartesian 3D EPI trajectory enabling motion correction in k‐space for functional MRI.

**Methods:**

The EPI “blades” of the 3D hybrid radial‐Cartesian EPI sequence, called TURBINE, are rotated about the phase‐encoding axis to fill out a cylinder in 3D k‐space. Angular blades are acquired over time using a golden‐angle rotation increment, allowing reconstruction at flexible temporal resolution. The self‐navigating properties of the sequence are used to determine motion parameters from a high temporal‐resolution navigator time series. The motion is corrected in k‐space as part of the image reconstruction, and evaluated for experiments with both cued and natural motion.

**Results:**

We demonstrate that the motion correction works robustly and that we can achieve substantial artifact reduction as well as improvement in temporal signal‐to‐noise ratio and fMRI activation in the presence of both severe and subtle motion.

**Conclusion:**

We show the potential for hybrid radial‐Cartesian 3D EPI to substantially reduce artifacts for application in fMRI, especially for subject groups with significant head motion. The motion correction approach does not prolong the scan, and no extra hardware is required. Magn Reson Med 78:527–540, 2017. © 2016 The Authors Magnetic Resonance in Medicine published by Wiley Periodicals, Inc. on behalf of International Society for Magnetic Resonance in Medicine. This is an open access article under the terms of the Creative Commons Attribution License, which permits use, distribution and reproduction in any medium, provided the original work is properly cited.

## INTRODUCTION

Three‐dimensional (3D) echo planar imaging (EPI) is attractive for high‐resolution functional MRI (fMRI), primarily due to gains in signal‐to‐noise ratio (SNR) efficiency as a result of using volumetric excitation and 3D Fourier encoding 1, 2, 3, 4. Other advantages of 3D imaging include the potential for higher parallel imaging acceleration factors due to acceleration along the second phase‐encoding direction, and the ability to achieve high isotropic resolution. The most commonly used 3D trajectory for fMRI is Cartesian multishot 3D EPI 1, for which readout planes are parallel. Other 3D trajectories that have been proposed for fMRI are echo volumar imaging 5, 6, 3D stack of spirals 7, and 3D radial 8. Radial‐based trajectories have a number of benefits for fMRI, including their potential for artifact reduction, which is crucial in 3D imaging for which sampling over several seconds can be required to form a complete image.

**Figure 1:**
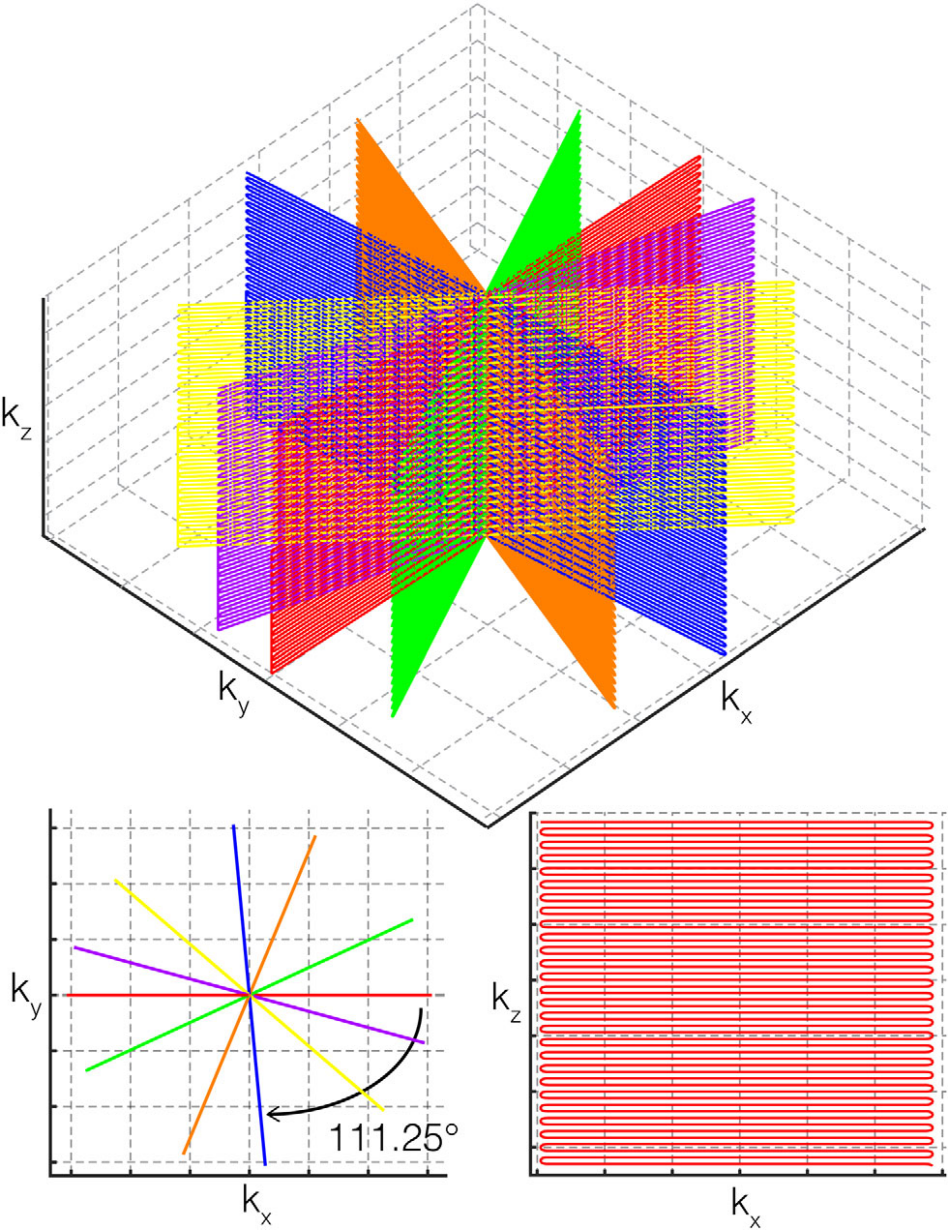
Diagram of the 3D hybrid radial‐Cartesian sampling scheme: Top schematic shows 3D k‐space of six shots. Each shot constitutes an EPI readout blade with phase‐encoding along k_z_; subsequent blades are rotated by approximately 111.25° about the k_z_ axis. Bottom row shows 2D projections of six blades onto k_x_‐k_y_ (left) and one example blade onto k_x_‐k_z_ (right).

The present work is based on a 3D hybrid radial‐Cartesian readout scheme (Fig. 1). The sequence, called Trajectory Using Radially Batched Internal Navigator *E*choes (TURBINE), was originally introduced for navigation of diffusion imaging 9 and recently began to be explored for fMRI 10, 11, 12. In the present implementation, EPI planes are rotated in a golden‐ratio angle scheme 13 about the phase‐encoding axis. This sequence has a number of benefits for fMRI:

1. The center of k‐space is crossed every shot, making the sequence self‐navigating for certain types of motion. Using a hybrid of radial and Cartesian sampling enables the potential for motion and physiological noise correction of radial imaging while maintaining encoding efficiency due to the use of EPI in the third dimension.
2. The golden‐angle update scheme allows flexible tradeoff between spatial and temporal resolution, which can be defined post‐acquisition. High spatial resolution enables the study of fine spatial features of functional neuroanatomy, as well as reduces dropout and partial volume artifacts. High temporal resolution improves the statistical robustness of fMRI signal estimates, enables study of blood oxygen level‐dependent (BOLD) signal dynamics, and can mitigate artifactual signal fluctuations such as aliased physiological noise.
3. The increased incoherence compared to purely Cartesian sampling makes this trajectory suitable for combination with k‐t acceleration schemes 14, 15, and adjusting the angle update scheme can modulate the amount of incoherence in the k‐t domain 16.

In this work, we focus on investigating the potential of TURBINE fMRI for motion correction. Bulk motion and other temporal fluctuations due to hardware instabilities and physiological noise have a detrimental effect on the ability to detect functional activity. Corruption by spurious fluctuations can require the exclusion of affected imaging volumes from analysis (scrubbing) or even the exclusion of entire subjects from studies, which reduces statistical power. Motion corruption also makes the clinical application of fMRI (e.g., presurgical planning, where robust single subject fMRI is required) challenging because many patients, especially pediatric patients, are unable to lie still for extended periods of time.

The standard motion‐correction approach in fMRI is optimized for rapid 2D imaging protocols, where it is assumed that subject motion is slow relative to the sampling window (tens of milliseconds for a single slice). In this case, the motion can be considered as inter‐volume only, which can be effectively dealt with by using image‐based registration in postprocessing 17. In 3D whole brain imaging, intra‐volume motion is non‐negligible because the sampling window is much longer (typically more than a second), which makes shot‐to‐shot k‐space inconsistencies more likely 18. Uncorrected, these k‐space inconsistencies can result in significant image artifacts. These include dropout due to phase cancellations, ghosting artifacts, and blurring, which reduces the effective spatial resolution of the acquisition. Therefore, in order to fully benefit from 3D imaging, these k‐space errors must be minimized or effectively removed.

Imaging strategies for correcting motion (see 19, 20 for reviews of the vast literature on this topic) in brain MRI can be classified based on when the correction is done, prospectively or retrospectively, and how the motion is estimated: 1) using external sensors (motion camera, respiration belt), 2) external navigation (acquiring additional data specifically to track fluctuations), and 3) self‐navigation. Motion cameras have great potential for both prospective 21, 22 and retrospective motion correction in fMRI, but they are not yet commonly available. External full 3D navigation can be problematic due to the lack of dead time in an optimized fMRI pulse sequence. Therefore, self‐navigated sequences are attractive for fMRI because they do not require the acquisition of any additional data. Examples for fMRI include PROPELLER fMRI 23, 24, self‐navigated spiral fMRI 25, and radial EPI 26 in 2D as well as 3D radial fMRI 8.

The goal of this work is to explore the utility of TURBINE for retrospective motion correction in fMRI in the presence of subject motion, exploiting its self‐navigating and flexible spatiotemporal resolution properties. We use the flexible temporal resolution of the readout to derive rigid body motion parameters from a spatially coarse, high temporal‐resolution navigator time series for adjustment of sampled data directly in k‐space. Furthermore, we exploit the self‐navigated properties of the readout to remove global phase fluctuations driven by subject motion and respiration.

## METHODS

### Pulse Sequence

**Figure 2:**
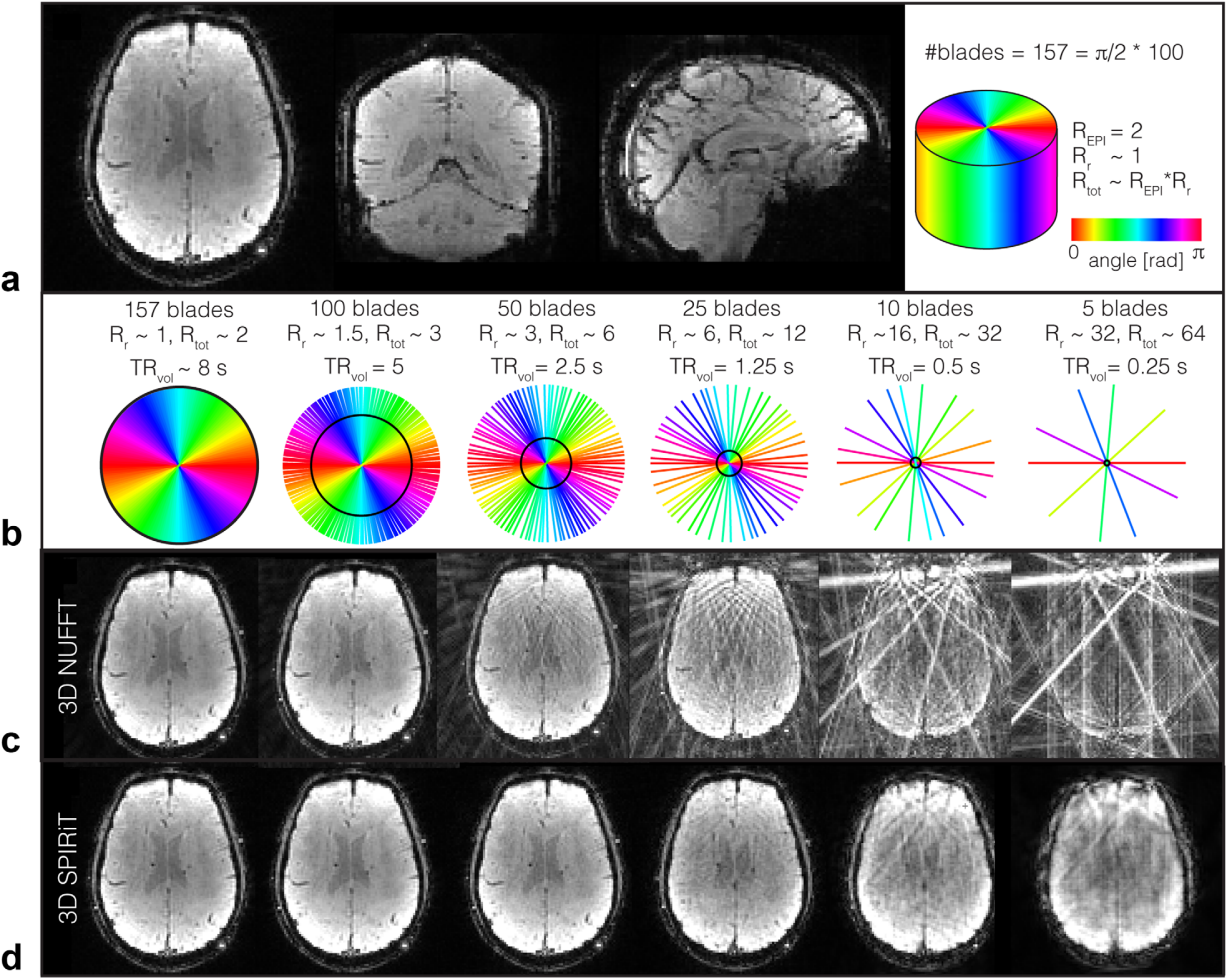
Flexible temporal resolution of hybrid radial‐Cartesian EPI: (a) Fully sampled (157 blades) reconstruction of an example volume. (b) K‐space plot of blades used in the reconstructions below, with the volume acquisition time and the corresponding undersampling factor relative to Nyquist criterion. The black circle indicates the point in k‐space at which the Nyquist criterion is still met. (c) Zero‐filled 3D NUFFT reconstruction, with aliasing artifacts introduced due to undersampling. (d) Reconstruction using the 3D SPIRiT parallel imaging algorithm.

The TURBINE pulse sequence implemented for this work is a 3D spoiled gradient echo sequence using a modified EPI readout. Selective excitation is used with the slab direction oriented along the phase‐encoding axis, and each repetition time (TR) one EPI readout plane (blade) is acquired (Fig. 1). In contrast to standard multishot 3D EPI, for which readout planes are parallel and offset in a second phase‐encoding direction, in TURBINE the EPI blades are intersecting and rotated about the EPI phase‐encoding axis. For this study, we chose an update angle based on the golden ratio: 180/1.618 ∼ 111.25°. This angle update scheme ensures near‐uniform angular coverage of k‐space for an arbitrary number of consecutive blades 13, which enables flexible post‐acquisition tradeoff between temporal and spatial resolution based on the Nyquist criterion, or alternatively variable temporal resolution at a fixed spatial resolution by varying the degree of undersampling in parallel imaging reconstruction (Fig. 2).

The blade phase‐encoding direction was chosen along the scanner head–foot direction (corresponding to the z‐gradient axis), which results in a Cartesian sampling distribution along k_z_ and radial distribution in k_x_ and k_y_. Having the phase‐encoding direction along a fixed axis results in constant distortion (compared to phase encoding along the radial direction). The trajectory samples a cylindrical k‐space volume, similar to a 3D stack‐of‐stars acquisition, with the difference being that the Cartesian axis is sampled in a single shot. This makes the sequence suitable for fMRI acquisition due to the long echo time (TE) required for optimal BOLD contrast and volume TRs on the order of a couple of seconds for whole brain imaging.

The sequence has conventional parallel imaging capabilities along the blade phase‐encoding direction (skipping k_z_ lines within the EPI readout), whereas the radial acceleration factor is defined post‐acquisition, depending on the number of shots binned into each 3D k‐space. Calibration datasets were acquired; these contained 200 blades, with each blade consisting of a sufficient number of EPI segments to match the echo spacing of the readout train. To minimize inconsistencies in the calibration data, all segments for a given blade are acquired consecutively 27, 28. Immediately after excitation, three nonphase encoded readout lines are acquired for EPI ghost correction.

### Motion Correction Concept

Motion correction with the proposed trajectory takes advantage of basic Fourier properties: an image space shift corresponds to a linear phase accumulation in k‐space, whereas a rotation in one domain is also a rotation in the other. Drifts and fluctuations in the static field B_0_ can also result in zero‐order phase offsets ${\Delta k}_{0}$, which can be caused by bulk subject motion and respiration.

In this work, we correct for rigid body motion only. Any rigid body transformation can be expressed as a rotation by the linear transform R, followed by a shift by the translation vector $\vec{T}=\left(\Delta x,\Delta y,\Delta z\right)$. In this case, the k‐space S of the moved object can be expressed with respect to the k‐space $S_{0}$ acquired of the object at the reference position:

(1) $$S\left(\vec{k}\right)=S_{0}\left(R\vec{k}\right)e^{-2\pi i(\vec{T}\cdot (R\vec{k})+{\Delta k}_{0}).}$$

The coordinates $\vec{k}={\left(k_{x},k_{y},k_{z}\right)}^{T}$ denote the nominal k‐space locations. Ideally, the rotation matrix, translation vector, and global phase offset would be known for all time points.

With the TURBINE trajectory, information from at least two blades needs to be combined to determine the rigid body motion in all three dimensions because each blade only provides information about the projection onto the plane in which it lies. There is likely to be an optimum number of blades expressing the tradeoff between estimation of motion parameters (favoring more blades) and the temporal accuracy of motion estimates (favoring fewer blades). In this study, we reconstructed a navigator image time series from a small number of blades and estimated motion parameters in image space by fitting a six‐parameter motion model. The motion was then corrected directly in k‐space according to Equation [1]. More details are provided in the image reconstruction section below.

### Data Acquisition

All data were acquired on a 3 Tesla (T) Siemens Prisma system using a 32‐channel head coil for reception. The objective of the in‐vivo experiments was to evaluate the extent to which the proposed k‐space trajectory and motion correction algorithm can correct for the whole range of possible subject motion. All five subjects were compliant healthy volunteers who moved very little unless instructed to do so. All acquisitions used TR/TE = 50/29 ms, with a blade EPI matrix of 100 × 76, and 2‐mm isotropic resolution, with R = 2 acceleration along the blade phase‐encoding direction. The readout bandwidth was 1852 Hz/pixel. For excitation, we used a binomial water‐excitation pulse with a flip angle of 15º (Ernst angle for gray matter), 3.84‐ms duration, and a time‐bandwidth product of 10. The slab thickness was set to 90% of the phase‐encoding field of view. At the end of every shot, the residual transverse magnetization was spoiled using 2π gradient spoiling along the x‐ and y‐axes, in addition to radiofrequency spoiling with a quadratic phase increment of 117° 29.

Three experiments were performed: 1) cued motions, 2) task fMRI with natural (deliberate but uncued) motion, and 3) task fMRI without explicit motion. Experiments 2 and 3 were performed in the same session with the order of the scans randomized.

1. Cued motion: Five subjects were visually cued to perform six different motions (x/y/z translations and rotations) in turn at specified intervals during a 130 s scan. The experiment was designed to evaluate the proposed correction in the full range of isolated rotations/translations.
2. Task fMRI with natural motion: Two subjects were instructed to be “fidgety” during a 300 s task fMRI experiment with visual stimulus and simultaneous motor task. The visual stimulus consisted of a 30 s OFF/ON 8 Hz flashing checkerboard stimulus, and subjects also were asked to perform bilateral finger tapping during the ON blocks.
3. Task fMRI without explicit motion: Repeat of experiment 2 with subjects instructed not to move.

We also performed a temporal SNR measurement for two subjects, consisting of two 10‐min resting scans each: one with natural motion (motion instructions as for experiment 2) and one with no motion (motion instructions as for experiment 3).

All subjects were instructed not to move in the first 20 s of all scans to avoid motion contamination of the parallel imaging calibration data. Subject breathing was measured using a respiratory bellows.

### Image Reconstruction and Motion Correction

All images were reconstructed offline in MATLAB (MathWorks, Natick, MA). One‐dimensional EPI Nyquist ghost correction 30 was applied on every blade using three non‐phase encoded navigator k‐space lines, which were acquired immediately after excitation. Coil compression (with virtual coils explaining ≥ 90% of the variance, typically yielding 10–12 coils) was used to accelerate the image reconstruction 31. Reconstruction of the undersampled data was performed using the k‐space–based parallel imaging methods GRAPPA 32 and SPIRiT 33. A 3D SPIRiT reconstruction was implemented by extending freely available 2D SPIRiT reconstruction software 33, using the Nonuniform Fast Fourier Transform (NUFFT) toolbox 34 to accommodate arbitrary k‐space sampling locations (including motion corrections, as described below). The 3D SPIRiT kernel calibration was performed on the fully sampled calibration data. The 2D GRAPPA kernels were calibrated for each blade angle using the closest blade in the calibration data, and GRAPPA was run on every blade prior to the 3D SPIRiT step. The final magnitude images were generated as a sum‐of‐squares combination of the individual reconstructed coil images.

**Figure 3:**
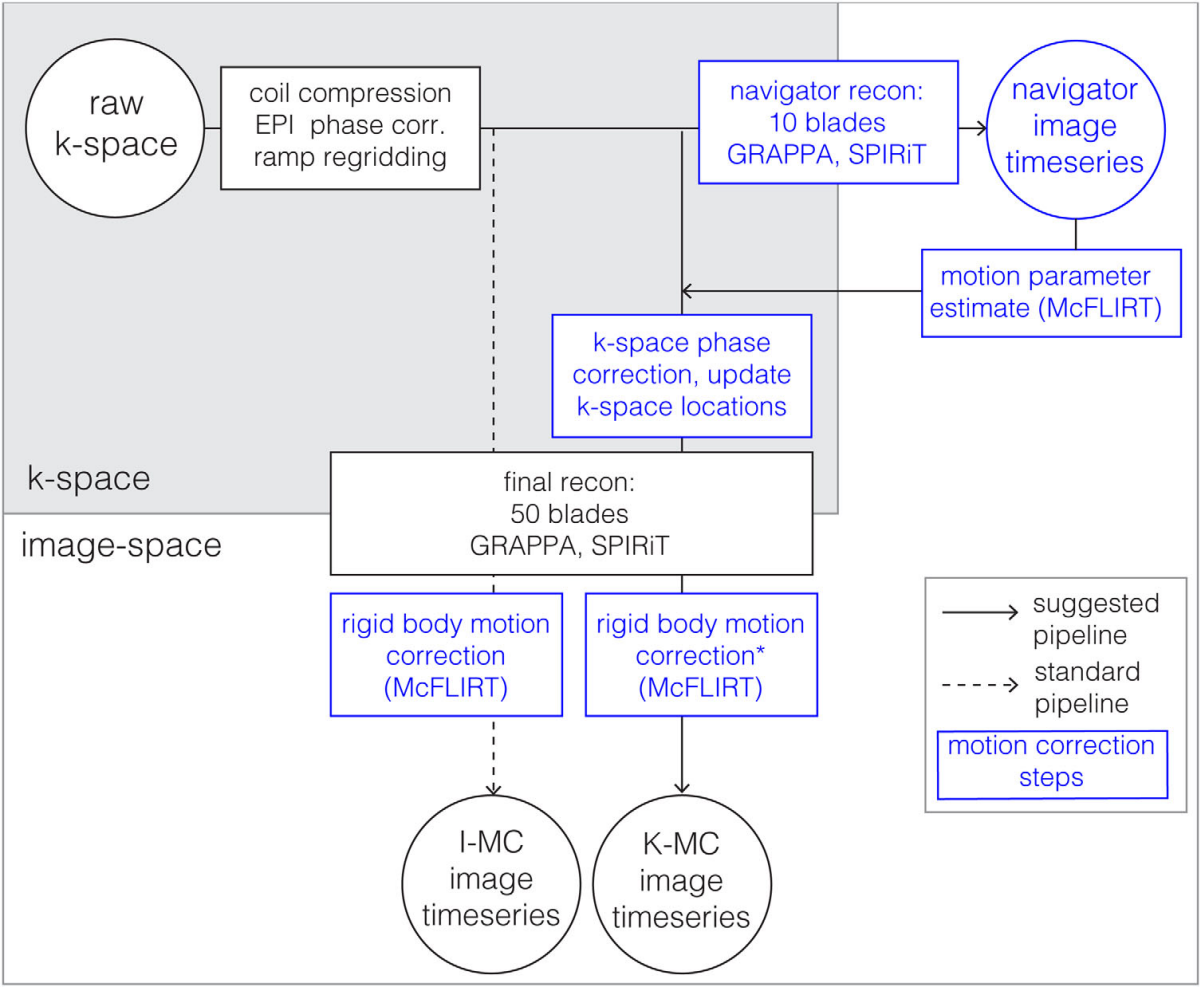
Overview of the motion correction and reconstruction process: K‐MC pipeline presented in this project (solid arrows) and standard approach I‐MC, which was performed for comparison (dashed arrows). The gray shaded area indicates the steps of the pipeline taking place in k‐space; all other steps operate in image space. Boxes and text in blue denote operations related to motion correction. The step marked with an asterisk is performed only for functional and image quality analysis.

An overview of the motion correction pipeline is presented in Figure 3. The high temporal‐resolution navigator time series was reconstructed using 10 EPI blades per navigator image (TR_vol_ = 0.5 s), Hanning‐filtered to reduce high spatial frequency artifacts. The rigid body motion was estimated in image space using the linear image registration tool MCFLIRT 17 in the FSL software library (FMRIB, Oxford, UK) relative to a reference volume, for which we chose the first volume of the time series. The motion parameters estimated from this registration were then fed back into the reconstruction to correct each blade individually. To generate motion estimates for each blade from the 10‐blade estimates, linear interpolation and spherical linear interpolation 35 were used for the translations and rotations, respectively. The zero‐order phase changes were estimated on a blade‐by‐blade basis (TR = 0.05 s) using the phase at the center of k‐space, and motion was corrected according to Equation [1]. To correct for rotations, k‐space sampling coordinates for each blade were rotated accordingly. The shifts and global phase offsets were removed by multiplication with the conjugated phase ramps. The final reconstructions using the corrected k‐space data were performed using 50 blades per image (TR_vol_ = 2.5 s), corresponding to a total undersampling factor of ∼6 (2 × 3.14) based on the Nyquist criterion for radial sampling (assuming regularly spaced blades). The correction pipeline was applied to all datasets, including those with no deliberate motion.

### Evaluation and FMRI Analysis

We compared the proposed motion correction (MC) pipeline to the standard approach using only image realignment (see Fig. 3). All datasets were processed with MCFLIRT to remove motion from the reconstructed image time series. When this was the only correction, we refer to this as I‐MC. Where we applied motion correction within the k‐space image reconstruction to remove reconstruction errors, we call this K‐MC; in this case, the final MCFLIRT alignments were expected to only remove small residual motions.

To quantify global inter‐volume motion in subjects before and after K‐MC in a single metric, we used a motion score 36 derived from MCFLIRT motion estimates, which corresponds approximately to a maximum possible displacement. Image quality reflecting the impact of the intra‐volume motion was quantified with an artifact score, which was defined as the root‐mean‐square of the difference image to a motion‐free reference volume. Task fMRI analysis was performed in FEAT (FSL) using no temporal filtering, spatial smoothing, or prewhitening. To account for residual autocorrelation, the resulting z‐statistic maps were corrected using mixture modeling 37 to ensure correct null distributions, with zero mean and unity standard deviation.

The functional data from the experiment without deliberate motion (experiment 3) was used to derive masks for the functional evaluation of experiment 2. Because this data was acquired in the same session, it should provide an estimate of the activation expected in a minimally motion corrupted acquisition to derive masks that neither favor K‐MC nor I‐MC. The masks were generated by intersecting thresholded (z ≥ 2.3) z‐stat maps from experiment 3, with hand‐drawn visual and motor cortex maps. All activation maps are displayed on a previously acquired T_1_‐weighted structural scan of the same subject.

## RESULTS

Figure 2a shows an example image acquired with the TURBINE sequence, reconstructed from k‐space, which was fully sampled in the radial direction (157 blades) and accelerated by a factor of 2 in the EPI plane. In contrast to the most commonly used EPI acquisitions, distortions are expected to manifest themselves along the z‐direction. A small amount of distortion can be seen in the frontal lobe in the sagittal view. More severe distortions only were observed when the volume had not been adequately shimmed (resulting in field inhomogeneity in the z‐direction).

Figures 2b‐d illustrate the tradeoff between temporal resolution and image quality offered by the radial golden‐angle sampling scheme. The undersampled zero‐filled reconstructions using the 3D NUFFT adjoint operator (Fig. 2c) for different numbers of blades contain aliasing artifacts characteristic of radial imaging. Reconstructing using the 3D SPIRiT parallel imaging algorithm (Fig. 2d) reduced these artifacts, with no visible artifacts up to total acceleration factors of R_tot_∼6. For the higher acceleration factors, an increase in image artifacts and noise can be observed. Compared to a fully Cartesian sampling scheme, TURBINE imaging is inherently slower by a factor π2. However, a higher amount of undersampling is tolerated due to the more incoherent radial sampling artifacts, which therefore allow similar volume acquisition times. We chose to use 50 blades (R_tot_∼6) for all final stage reconstructions, allowing whole brain coverage with 2‐mm isotropic resolution in 2.5 s.

**Figure 4:**
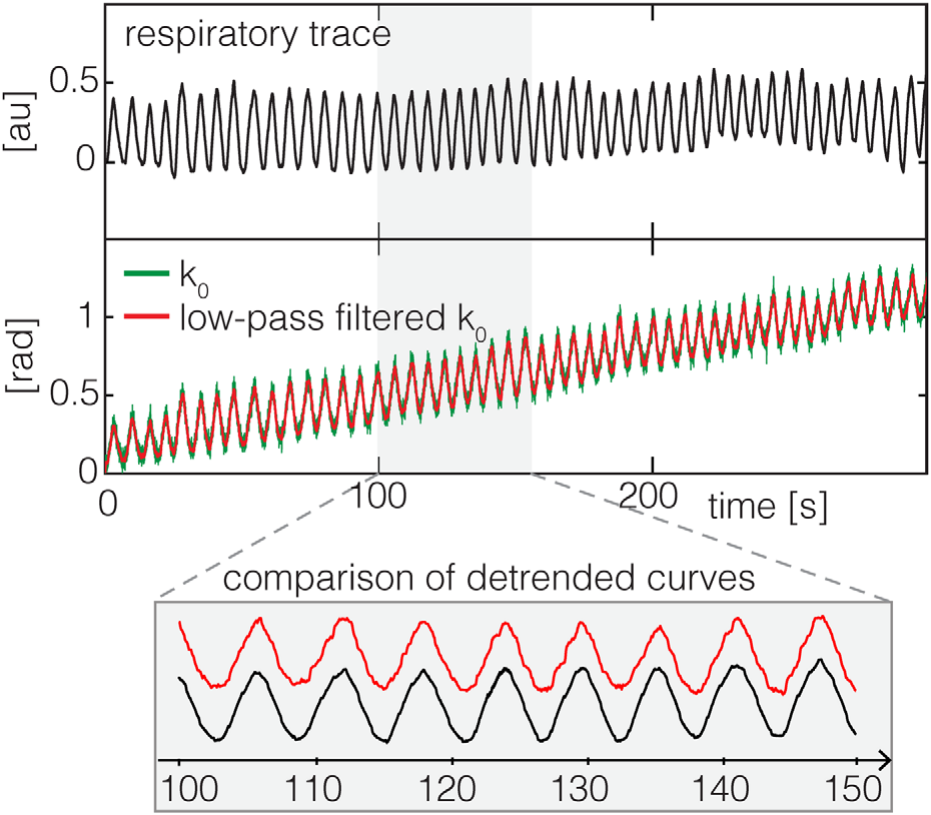
Global phase correction: Zero‐order phase term for example dataset, with no motion displayed in green and low‐pass filtered version in red. Zoomed plot below shows comparison of detrended global phase estimate with the corresponding respiratory trace, measured with a respiratory bellow.

The global (zero‐order) phase offset k_0_ reflects variations in B_0_ (Fig. 4). It tracks the measured subject respiration (red lines), which can be seen through comparison to the externally measured respiratory trace (black lines), and captures the slow B_0_ drift over the course of the acquisition (trend in red and green lines). In the presence of large motion, jumps in the global phase term can be observed.

### Cued Motion Experiment

**Figure 5:**
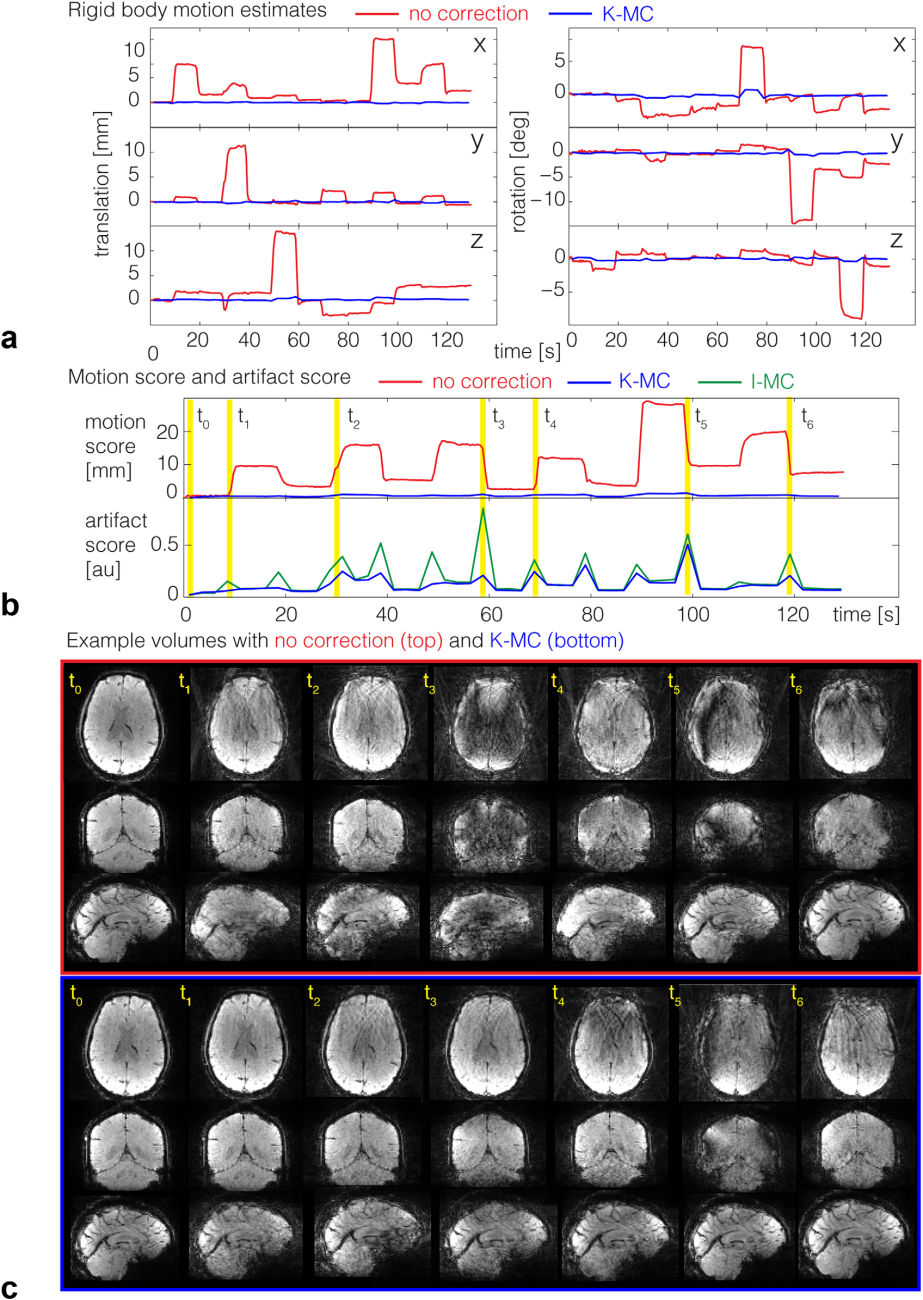
Cued motions (experiment 1): (a) Translation and rotation estimates prior to correction for a representative subject displayed in red, residual motion after K‐MC (detected using MCFLIRT) in blue. The motion estimates before correction are calculated from the navigator time series and used for correction, whereas the residual motion is measured on the final image time series. (b) Motion score summarizing motion prior to correction (red) and residual motion after K‐MC (blue) in top panel; artifact score on final images using K‐MC (blue) or I‐MC (green) in bottom panel. (c) Example images, showing transversal, coronal, and sagittal slices of uncorrected (top) and K‐MC (bottom) volumes for seven time points. Time points marked with yellow lines in (b): t_0_ is the first volume containing no motion; t_1_‐t_6_ are example volumes at the different motion transitions.

**Figure 6:**
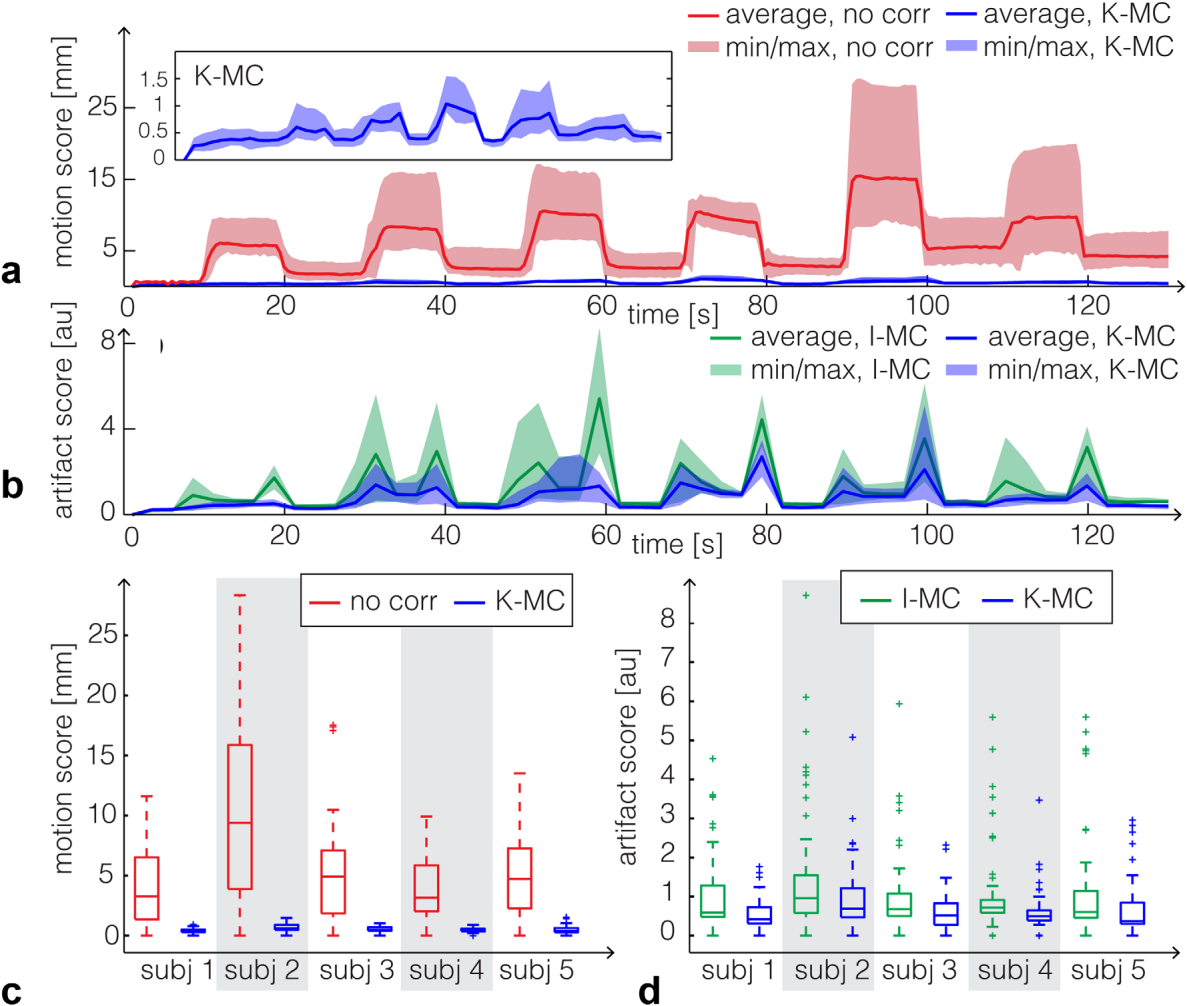
Cued motions (experiment 1): (a) motion score and (b) artifact score over time summarized for all subjects. The solid line shows the average over all subjects, and the shaded area denotes the minimum/maximum value over all subjects. The inset shows the motion score after K‐MC on an inflated scale. (c) Box‐whisker plot of the motion score before correction and after K‐MC. This corresponds to an overall reduction in motion score of 90%. (d) Box‐whisker plot of the artifact score, comparing K‐MC to I‐MC, showing a reduction of over 40%.

The results of the cued motion experiment are shown in Figures 5 and 6. The motion estimates derived from the navigator time series (red lines) and those from the final reconstruction using K‐MC (blue lines) are shown for a representative subject in Figure 5a. The subjects performed translations in x (left–right), y (anterior–posterior), and z (head–foot), followed by rotations about x (nod), y (tilt head), and z (shake head). All subjects executed the motions correctly, but most contained a combination of movements (e.g., x translations accompany rotations about y and z in Figure 5a) because some of the motions are difficult to execute in an isolated fashion. The low‐motion parameters and score after K‐MC (blue lines) in Figures 5a‐b indicate very low residual motion. These values demonstrate the efficacy of the K‐MC reconstruction, which is expected to remove bulk motions as well as I‐MC does. The artifact scores, as well as visual inspection of the images, were used to assess how effectively K‐MC reduces artifacts compared to I‐MC, which cannot remove within‐volume inconsistencies.

The artifact score (Fig. 5b) was reduced substantially following K‐MC (blue line) compared to I‐MC (green line). Many volumes of the uncorrected datasets contain severe artifacts (Fig. 5c), such as radial streaking artifacts and dropout artifacts, caused by phase inconsistencies from blade to blade at the k‐space center where the trajectories intersect. The artifacts are removed or reduced in the K‐MC time series. Some volumes, especially those for which large rotations occurred, display residual artifacts (e.g., time point t_5_), although in all cases these artifacts are reduced in K‐MC compared to I‐MC. A movie of an example cued‐motion dataset comparing uncorrected, K‐MC, and I‐MC is included in Supporting Video 1.

The results of the cued motion experiments for all five subjects are summarized in Figure 6, with plots 6a‐b showing plots of motion scores over time and Figures 6c‐d showing box‐whisker plots of motion and artifact score for all time points for each individual subject. The plot in Figure 6a displays the average (bold line) and minimum and maximum (shaded area) motion scores over all subjects, with no correction (red) and using K‐MC (blue). The average motion score for the motion estimates before correction was approximately 15 mm, and the maximum exceeded 25 mm. After K‐MC, the largest residual motion score was 1.5 mm (inset in Fig. 6a). Residual translations along any axis did not exceed 0.7 mm and are below 0.5 mm for all but one subject. The maximum residual rotation along any axis was 1° for one subject and was much lower for all other subjects. Figure 6b equivalently shows the artifact score, which is generally reduced substantially for K‐MC compared to I‐MC. However, for certain subject motions (e.g., at t = 100 s), there are substantial residual artifacts. Averaged over all subjects and time points, the motion scores were reduced by 90.0%, and artifact scores were reduced by 40.2%. The box‐whisker plots for the individual subjects (Figs. 6c‐d) show large differences between motion amplitudes and artifact level. In no cases were artifacts observed to increase following correction, either through visual inspection or based on artifact score.

### fMRI With Natural Motion

**Figure 7:**
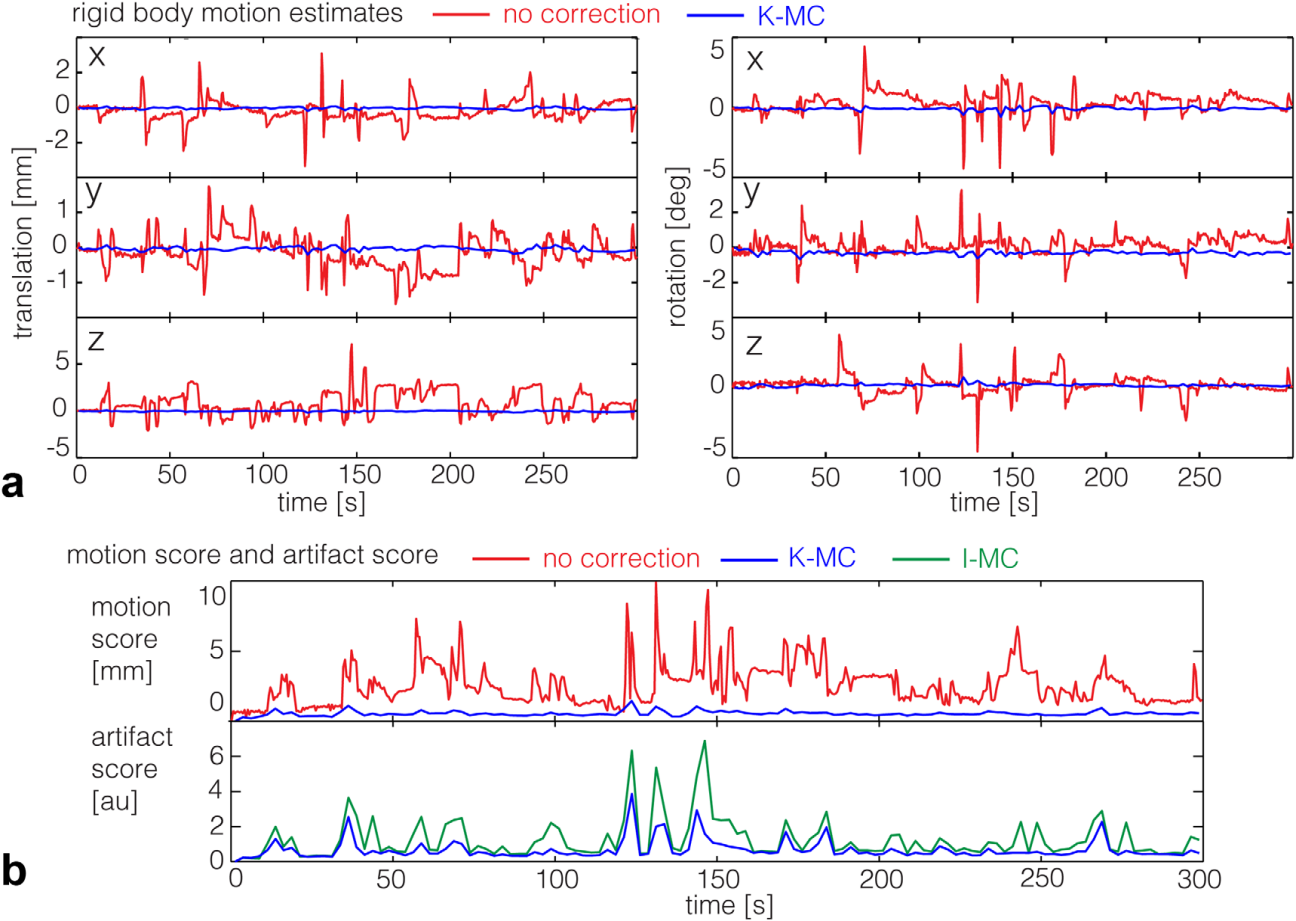
fMRI with natural motion (experiment 2): Translation and rotation estimates based on the navigator time series (red) for a representative subject for the fMRI experiment with deliberate but uncued motion. Residual motion after performing motion correction shown in blue.

In the natural motion experiment, the subjects moved using all degrees of freedom (example subject in Fig. 7a), but z translations and x rotations had greater magnitude and were more frequent than the other motions. Presumably, this reflects movement constraints by the coil and padding along the other directions and/or the most natural types of head motion. The translations were within a range of 10 mm, and rotations did not exceed a range of 10°, both larger than would normally be expected of naïve but compliant subjects. Using K‐MC, the average motion scores were reduced by 90% and 76%, and the average artifact scores were reduced by 41% and 46% for subjects 1 and 2, respectively, compared to I‐MC (Fig. 7b). A movie of a natural motion dataset is provided in Supporting Video 2.

**Figure 8:**
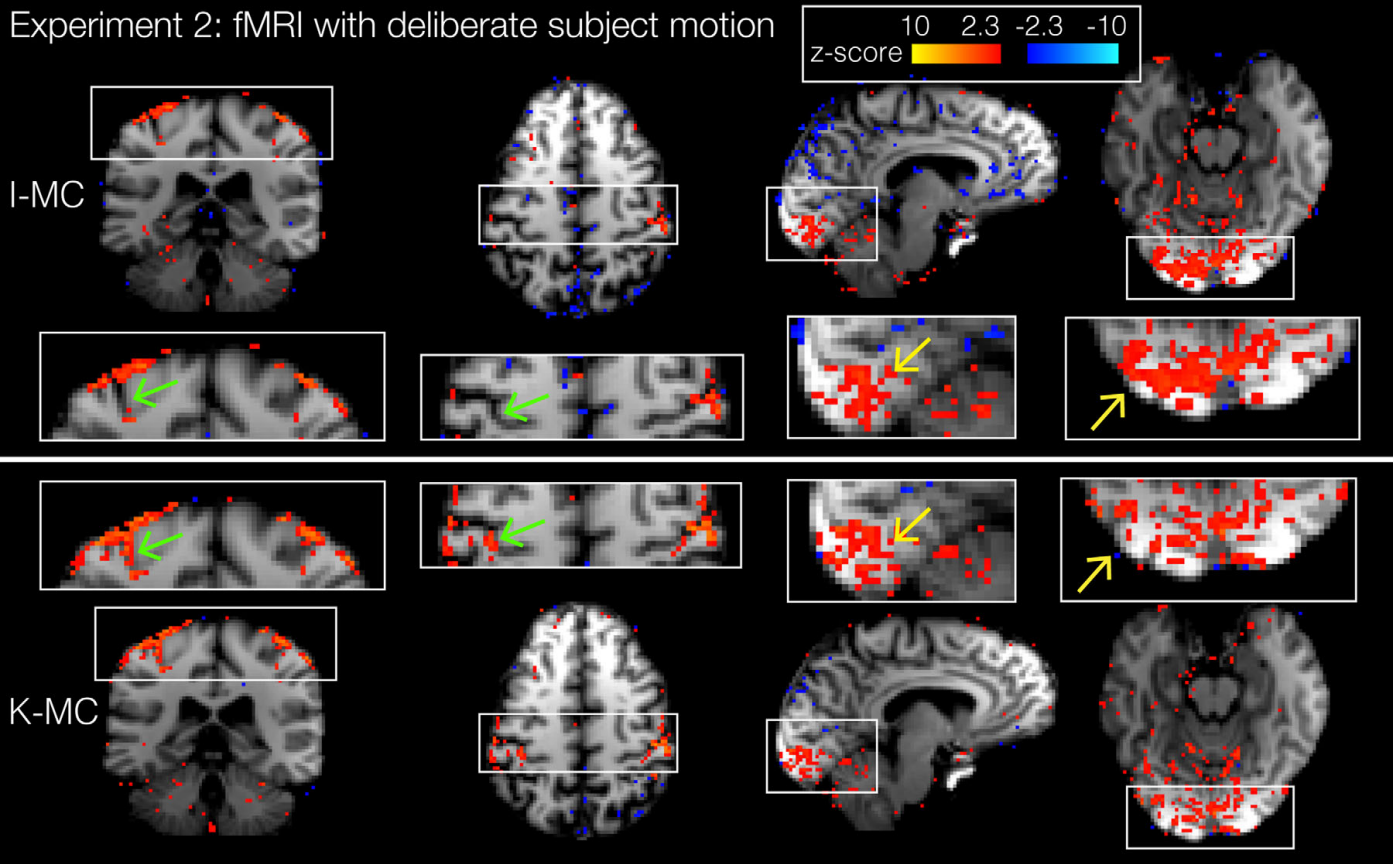
fMRI with natural motion (experiment 2): Activation maps for subject 1 using I‐MC (top) and K‐MC (bottom). Green arrows in zoomed images indicate examples of activation recovered using K‐MC. Yellow arrows highlight reduction of misplaced activation when using K‐MC.

**Figure 9:**
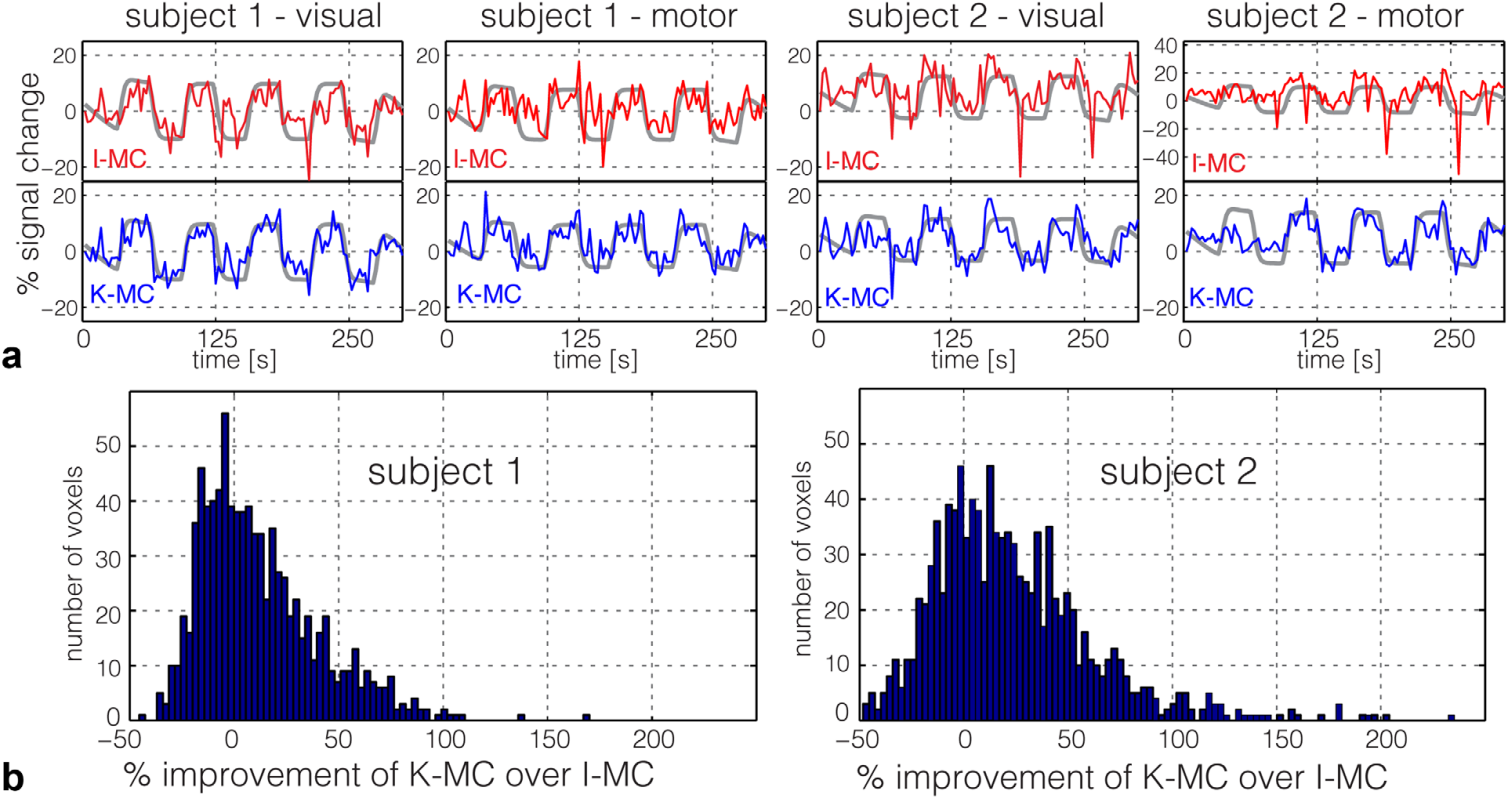
fMRI with natural motion (experiment 2): (a) Average time courses for motor and visual activation for both subjects using a high z‐stat ROI based on the activation maps from the data, with no explicit motion. Plots are displayed for K‐MC (blue) and I‐MC (red), with the FSL model underlaid in gray. (b) Histograms of percent improvement of K‐MC over I‐MC for active (z ≥ 2.3 in both K‐MC and I‐MC) voxels in visual and motor cortex ROI.

Figures 8 and 9 show the result of the functional evaluation of the visual‐motor task fMRI experiment with natural motion. The activation maps for subject 1 (Fig. 8) show visual and motor activation for both K‐MC and I‐MC. In the motor cortex, the activation for K‐MC localizes to the characteristic omega‐shape of the hand region of the precentral gyrus and also follows the folding of the central sulcus in the coronal view (hook‐shape). This is partially missing in the I‐MC activation maps, indicating that the K‐MC reconstruction recovered some of the activation that had been lost due to motion (green arrows). Some of the visual activation in the I‐MC data crosses white‐matter boundaries (yellow arrows), suggesting that the activation is artifactual or misplaced. In the maps for K‐MC (see zoomed subplots of the sagittal and transversal view in Fig. 8), the activation follows the sulcal anatomy more accurately. The blue color scheme for z‐stats represents artifactual negative correlations, likely driven by stimulus‐correlated motion, which are reduced in K‐MC compared to I‐MC (visible in sagittal view in Fig. 8).

For quantitative analysis of the z‐stats, we derived regions of interest (ROIs) from the data with no explicit motion (as an approximation to a ground truth estimate for the correct activation). The number of voxels with activation (z ≥ 2.3) in visual and motor cortex ROIs increased respectively by 16% and 113% for subject 1, and by 5% and 41% for subject 2. The average z‐stats in the same ROIs increased by 13% and 54% for subject 1, and by 18% and 40% for subject 2.

Figure 9a plots time series for using refined visual and motor masks that include only the 100 most active voxels in each mask (based on the low‐motion functional reference scan). The time courses follow stimulus design more clearly for K‐MC time courses than for I‐MC. We evaluated percent improvement of the z‐stats in the combined visual and motor ROI with the additional conditions of voxels included, having z ≥ 2.3 both for the I‐MC and K‐MC dataset (see histogram in Fig. 9b). For both subjects, the histograms exhibit a strong skew, indicating that the z‐stats for the majority of voxels in the ROI are improved.

### fMRI With No Explicit Motion

**Figure 10:**
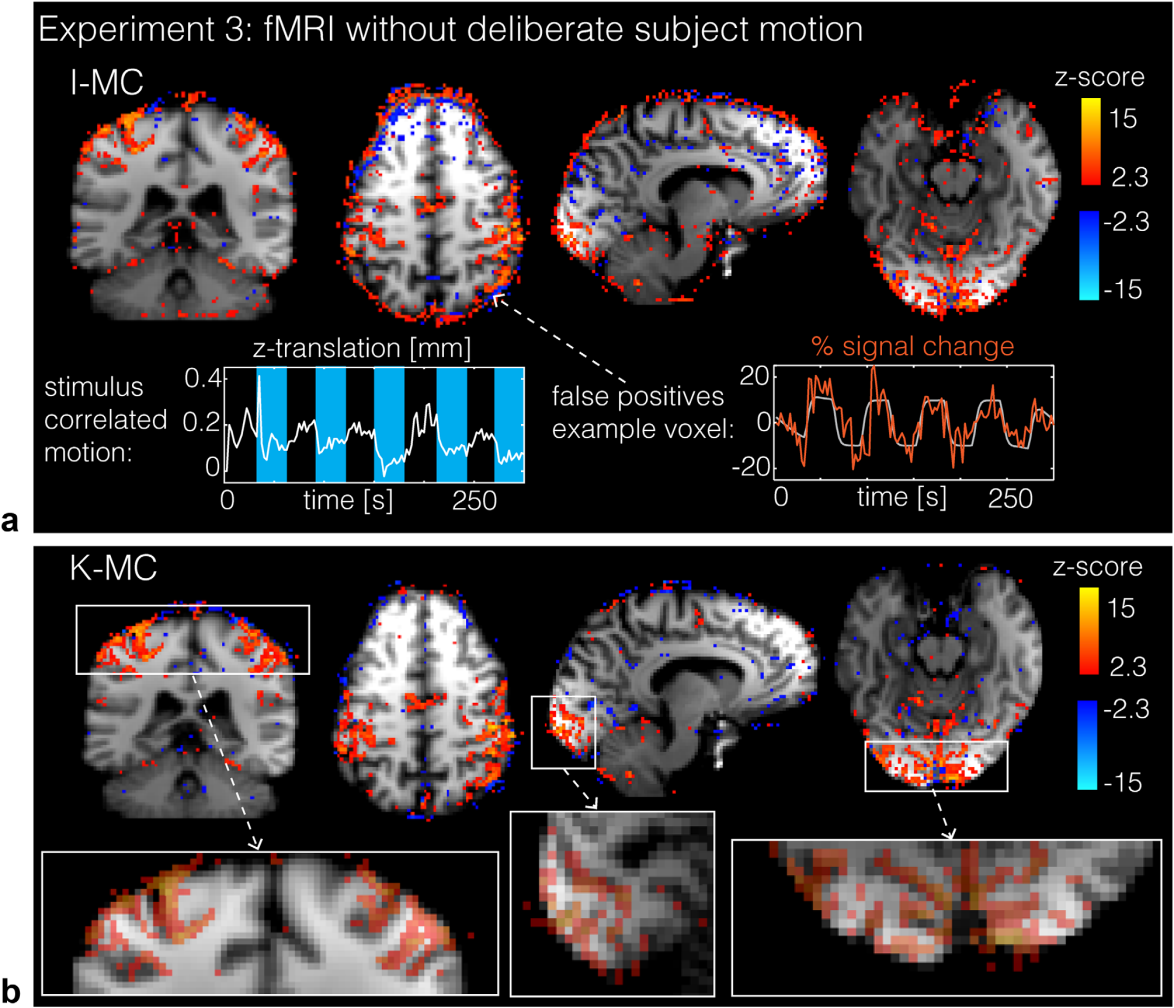
fMRI with no deliberate motion (experiment 3): Activation (z‐stat) maps for an example subject with a small amount of (non‐deliberate) motion. (a) I‐MC z‐stats maps containing a large amount of false positives, primarily at tissue boundaries. The false positives result from stimulus‐correlated motion, as illustrated by insets (motion in the z‐direction and time course for an example edge voxel). (b) K‐MC z‐stats maps, with reduced false positives. Zoomed activation maps below display positive z‐stats maps overlaid (semi‐transparent) on structural image to show correspondence of activation with gray matter architecture.

In the visual‐motor task fMRI experiment with no explicit motion, subjects moved only minimally: the maximum motion score did not exceed 2 mm for either of the subjects; and the average motion score was 0.5 mm and 1 mm for subjects 1 and 2. The z‐stat maps (Fig. 10) for subject 1 with I‐MC show activation in the visual and motor cortices typical for the task used, but also a substantial amount of false positives (active voxels at boundaries of the head and isolated active voxels within structures that are not expected to be active in the task). Analysis of the motion estimates showed that both subjects moved (small amounts) in a stimulus‐correlated fashion, clearly visible in the z‐translation motion estimate (Fig. 10a), which is likely due to the subject moving unintentionally when finger tapping. The K‐MC reconstruction substantially reduces motion‐induced false positives, with the activation maps showing specific activation following the gray‐matter outlines in the visual and motor cortex (Fig. 10b).

Finally, the temporal SNR in the experiments with natural motion was averaged over the entire brain. Temporal SNR (tSNR) increased by 28% (from 8.3 to 10.7) and 36% (from 9.9 to 13.5) for subjects 1 and 2, respectively, when using K‐MC over I‐MC. For the same scan with no deliberate motion, the whole‐brain averaged tSNR was 31.0 and 27.9 for subjects 1 and 2, respectively. In ROIs defined for the visual and motor cortex areas, the tSNR was 40.1 and 39.8, respectively, for subject 1; and it was 28.9 and 37.7, respectively, for subject 2 (see Sup. Fig. S1 for tSNR maps).

## DISCUSSION

The TURBINE trajectory used in this work was originally designed for navigated diffusion imaging 9. The combination of EPI with radial imaging has also recently been used for perfusion imaging 38. Preliminary reports using TURBINE‐like trajectories for FMRI have been made by our group and other groups, exploring golden‐angle increments 11, 12, 39, use at ultrahigh‐field 10, and combination with k‐t acceleration 14. In this work, we have demonstrated the motion correction capabilities of the TURBINE trajectory for fMRI, even in the presence of severe motion. The approach requires no designated hardware and no separate navigator scans; therefore, it is highly compatible with fMRI, for which we require short‐volume TR and re‐acquisition is not possible. We used the intrinsically flexible temporal resolution of the k‐space acquisition to derive motion estimates from the reconstruction of a high temporal‐resolution navigator time series. The approach to derive motion parameters from navigator images has been used for a number of different applications 40, 41.

Using TURBINE with K‐MC, most of the bulk motion was corrected, and artifacts were reduced substantially for all motion‐corrupted datasets. We were able to improve functional results both in datasets with very large subject motion and more subtle stimulus‐correlated motion, as reflected by recovered functional activity and reduced false positives. Residual motion artifacts (signal dropout and radial streaking) were observed mainly for the most highly corrupted volumes (i.e., those with very high artifact score before K‐MC). This could be due to the rigid body‐motion estimate being less accurate as a result of high levels of artifact in the navigator volumes, which are reconstructed at a relatively coarse 0.5 s temporal resolution. A disadvantage of the image‐based motion detection approach is that the accuracy depends on the spatial resolution and image quality of the input time series, leading to a tradeoff between motion estimate fidelity and temporal resolution of the motion estimates. The 0.5 s windows used here for navigator reconstruction limit the temporal fidelity of the motion estimates, but in future work we will explore sliding window filters (e.g., temporal hourglass 42), which have been shown to improve the temporal resolution of motion detection. Residual errors might also come from variation in distortion and coil sensitivity when the head moves within the B_0_ field and the coil, invalidating the calibrated GRAPPA or SPIRiT kernels. Further inconsistencies might be driven by changes in the MRI signal due to the large‐scale movements present in the data, which K‐MC cannot correct for. For very heavily corrupted datasets, the tSNR remains substantially lower than for an uncorrupted dataset even after correction, due to residual image artifacts.

A direct estimation of the motion parameters in k‐space has been shown to work well in 2D radial sequences 43. In previous work 12, we demonstrated robust estimation of shifts along the z‐direction, directly in k‐space on a blade‐by‐blade basis when motion only along this direction is present. This single‐blade estimate becomes inaccurate during simultaneous rotations, but combining information across two or more blades provides enough information to allow all six motion degrees of freedom to be estimated. In future work, we will explore whether a k‐space fit of all motion parameters or further improving the temporal resolution of the image‐based estimates (as suggested above) is more promising to improve the motion correction results. We will also consider estimation of motion directly within the iterative reconstruction algorithm by incorporating the motion into the reconstruction model 44, 45.

Due to the different sampling strategy in TURBINE, changes in B_0_ (creating phase inconsistencies between blades) and time‐varying aliasing (compared to using fixed sampling) could reduce tSNR. The tSNR values in the datasets with negligible motion are within the range reported for 2D and 3D EPI in the literature 1, 2, 3, 4, 46, 47, 48, 49; see Supporting Tables S1 and S2 for a summary of literature tSNR values. TURBINE could be acquired with a fixed angle scheme to have the same aliasing pattern for all imaging volumes, but this version would lose full flexibility of the temporal resolution. Our decision to use the continuous golden‐angle approach was motivated by the desire to demonstrate flexible temporal resolution, enabled by the low levels of observed aliasing in well‐behaved subjects. It would be informative to compare the tSNR values calculated here to SNR to determine the signal stability. Unfortunately, the SNR is difficult to assess, and calculation would need to account for receive coil inhomogeneity, parallel imaging noise amplification, spatial noise autocorrelation due to the non‐Cartesian trajectory, and the presence of residual streaking artifacts. For these reasons, SNR calculated with an ROI‐based method is unlikely to provide meaningful results; we focus our analyses to tSNR, which is the more relevant quantity for fMRI.

The sampling SNR efficiency of non‐Cartesian trajectories is intrinsically lower than uniform sampling 50, but the aliasing artifacts are more incoherent; therefore, higher undersampling factors are tolerated. Further increasing the robustness to undersampling is important to allow TURBINE to compete with fast Cartesian 3D EPI methods, some of which are capable of achieving whole brain coverage at 2 mm isotropic resolution in under a second 48. If a Cartesian sampling strategy could acquire the entire 3D k‐space as fast as TURBINE navigators, the benefits of TURBINE will solely be due to correction of global phase inconsistencies. However, the fastest theoretical 3D navigator in TURBINE consists of two blades; with sequence refinements and integration of the motion detection into the image reconstruction pipeline, improved temporal resolution of motion detection should be achievable.

The aliasing properties of the trajectory could be improved by ensuring more distributed k‐space samples. For in‐plane acceleration, the skipped lines could be alternated from shot‐to‐shot (e.g., for R = 2, every second blade acquires even lines and the others acquire odd lines). It would also be beneficial to include controlled aliasing in parallel imaging (CAIPI) 51, 52 gradient blips (shifting k‐space samples perpendicular to the blade orientation) because 3D EPI‐CAIPI 48, 53, 54 has been shown to have superior undersampling properties and high tSNR. For higher spatial resolution, an asymmetric readout might be beneficial to allow for short‐blade TR and shorter TEs for 7T imaging.

The benefit of 3D in terms of SNR efficiency primarily relates to the ability to rapidly repeat volumetric excitations, whereas 2D methods—including simultaneous multislice (SMS)—must cycle through slices before a given slice is excited again. The Achilles heel of 3D EPI is that it is segmented, making it sensitive to physiological noise and motion, whereas 2D EPI or SMS are relatively insensitive to motion (although still susceptible to spin‐history artifacts 55). TURBINE has the potential to correct for motion and therefore combine high frame rate (conferring statistical benefits) with high SNR efficiency. The absolute stability of the approach, in terms of tSNR, is what ultimately matters. It will be important to systematically evaluate whether 3D TURBINE with K‐MC provides better fMRI results than a Cartesian 3D EPI or 2D (multiband) EPI acquisition with the best motion correction that can be done on all datasets. In future work, we hope to assess under what conditions (e.g., resolution, amount of motion) TURBINE confers the most benefit. We expect this to be high‐resolution fMRI at ultra‐high field, where 3D methods provide larger advantages over 2D and high resolution requires relatively long and therefore motion‐sensitive acquisitions. For higher spatial resolution, improvement of the resolution of the navigator time series will be important in order to guarantee the same motion correction accuracy.

For the reconstruction of undersampled data, we applied GRAPPA on each blade prior to reconstructing the entire volume with 3D SPIRiT because this removed aliasing (in the blade phase‐encoding direction) more robustly than 3D SPIRiT alone. The image reconstruction was not a primary focus of investigation in this study; other non‐Cartesian parallel imaging methods, such as CG SENSE 56 or ESPIRiT 57, can be considered instead of SPIRiT. For example, we have already demonstrated the use of rank‐constrained k‐t accelerated reconstructions using this trajectory 14. The reconstruction and motion correction of an entire fMRI dataset currently takes a few hours, but this could be sped up and automated (e.g., using GPU‐based software).

In this study, we used compliant subjects to study the ability to correct for motion during the main phase of data acquisition. These subjects were specifically directed not to move during parallel imaging calibration acquisitions (first ∼20 s of scan) because motion‐corrupted calibration data can result in poor image reconstructions. If the subject cannot lie still for this amount of time, the protocol could be modified to be autocalibrating by acquiring a fully sampled central region of k‐space for every blade, or altering the phase‐encoding steps such that a fully sampled k‐space is acquired after some number of blades. In that case, any motion‐free segment of data could be selected to perform the parallel imaging calibrations. This would have further advantages because non‐autocalibrated parallel imaging in a motion‐corrupted dataset is problematic in general.

The protocol run in this study was observed to induce low levels of peripheral nerve stimulation (PNS). The PNS is within the scanner safety limits and of similar magnitude, as for standard EPI using the y‐gradient (anterior‐posterior direction readout) for the readout. The PNS could be reduced by changing the gradient axes used in the readout direction or by increasing the echo spacing (resulting in a longer acquisition).

In order to evaluate detection of activation in the presence of motion, we chose a relatively simple and robust task involving a visual stimulus with simultaneous finger tapping. This task was simple enough to enable subjects to simultaneously conduct deliberate motions. However, there may be some downsides to this experimental design. Visual activation in the occipital lobe is located at a relatively fixed pivot point, making this area particularly robust to subject motion. Similarly, subjects' motor cortices were engaged in both finger tapping task and cued motions, which could lead to poor task compliance and/or interactions between task and artifact. It will therefore be useful to consider more realistic settings, including higher level tasks and more realistic motions.

A further advantage of this 3D radial‐Cartesian trajectory is its more incoherent sampling compared to fully Cartesian approaches, making it suitable for sophisticated reconstructions that build on concepts in data compression. For example, the trajectory presented here has been successfully used for fMRI, using k‐t matrix completion with fixed‐rank constraints to achieve high temporal resolution 14, 15. In future work we hope to combine the motion correction presented in this work into these k‐t accelerations, which may improve the quality of fixed‐rank reconstructions given that motion can violate low‐rank assumptions.

## CONCLUSION

We have demonstrated that the hybrid radial‐Cartesian EPI is suitable for motion correction in fMRI. Using the flexible spatio‐temporal resolution of this sampling strategy, we were able to correct data corrupted by severe subject motion and improve recovery of fMRI activation. Unlike many other methods for motion correction, this approach does not prolong scan time and no extra hardware for motion tracking is required.

## Supporting information


**Fig. S1**. TURBINE tSNR maps: Maps of temporal SNR for two subjects with negligible amounts of subject motion (only I‐MC was performed on this data). The whole‐brain averaged tSNR was 31.0 and 27.9 for subjects 1 and 2 respectively. In ROIs defined for the visual and motor cortex areas the tSNR was 40.1 and 39.8 respectively for subject 1 and 28.9 and 37.7 for subject 2.
**Table S1**. Example 3D EPI tSNR values (for grey matter ROIs) reported in the literature. The studies marked with an asterisk * also report tSNR for 2D EPI protocols, which are included in Supporting Table S2. When comparing values, differences in parameters such as field strength and voxel volume, as well as their impact on physiological noise need to be considered. The last column contains the volume TR as well as the parallel imaging acceleration factor (AF) and amount of partial Fourier (PF) if applicable. When the study used physiological noise correction (Lutti 2, Jorge 3 and Narsude^48^), the tSNR values using physiological noise correction were included. It should be noted that many of these protocols focused on tSNR efficiency (i.e. tSNR per unit time) rather than raw tSNR.
**Table S2**. Example 2D EPI tSNR values (for grey matter ROIs) reported in the literature. The studies indicated with an asterisk * also report tSNR for 3D EPI protocols, which are included in Sup. Table S1. See also the caption of Sup. Table S1 for further explanation.

**Video S1**. Video of cued motion (experiment 1): Example subject performing cued motions; showing sagittal (top) and transversal (bottom) view of uncorrected (left) K‐MC (middle) and I‐MC (right) image time‐series. The I‐MC images are blurred due to the interpolation applied for image‐based MCFLIRT registration. In the K‐MC images the resolution is preserved and the artifact level is reduced compared to I‐MC. Some volumes of the K‐MC time series contain residual artifacts.

**Video S2**. Video of natural motion (experiment 2): Example subject moving naturally (deliberate but uncued) during task fMRI scan; showing sagittal (top) and transversal (bottom) view of uncorrected (left) K‐MC (middle) and I‐MC (right) image time‐series.
