# Supplementary material for: Motion correction for functional MRI with three‐dimensional hybrid radial‐Cartesian EPI
Source: Magn Reson Med. 2016 Sep 8;78(2):527–40. doi: 10.1002/mrm.26390 (PMC5516130; doi:10.1002/mrm.26390)
Supplement: Supplementary file 1 — Fig. S1. TURBINE tSNR maps: Maps of temporal SNR for two subjects with negligible amounts of subject motion (only I‐MC was performed on this data). The whole‐brain averaged tSNR was 31.0 and 27.9 for subjects 1 and 2 respectively. In ROIs defined for the visual and motor cortex areas the tSNR was 40.1 and 39.8 respectively for subject 1 and 28.9 and 37.7 for subject 2. Table S1. Example 3D EPI tSNR values (for grey matter ROIs) reported in the literature. The studies marked with an asterisk * also report tSNR for 2D EPI protocols, which are included in Supporting Table S2. When comparing values, differences in parameters such as field strength and voxel volume, as well as their impact on physiological noise need to be considered. The last column contains the volume TR as well as the parallel imaging acceleration factor (AF) and amount of partial Fourier (PF) if applicable. When the study used physiological noise correction (Lutti 2, Jorge 3 and Narsude48), the tSNR values using physiological noise correction were included. It should be noted that many of these protocols focused on tSNR efficiency (i.e. tSNR per unit time) rather than raw tSNR. Table S2. Example 2D EPI tSNR values (for grey matter ROIs) reported in the literature. The studies indicated with an asterisk * also report tSNR for 3D EPI protocols, which are included in Sup. Table S1. See also the caption of Sup. Table S1 for further explanation. [file MRM-78-527-s001.docx]

Supporting figure:

**Sup. Fig S1.** TURBINE tSNR maps: Maps of temporal SNR for two subjects with negligible amounts of subject motion (only I-MC was performed on this data). The whole-brain averaged tSNR was 31.0 and 27.9 for subjects 1 and 2 respectively. In ROIs defined for the visual and motor cortex areas the tSNR was 40.1 and 39.8 respectively for subject 1 and 28.9 and 37.7 for subject 2.


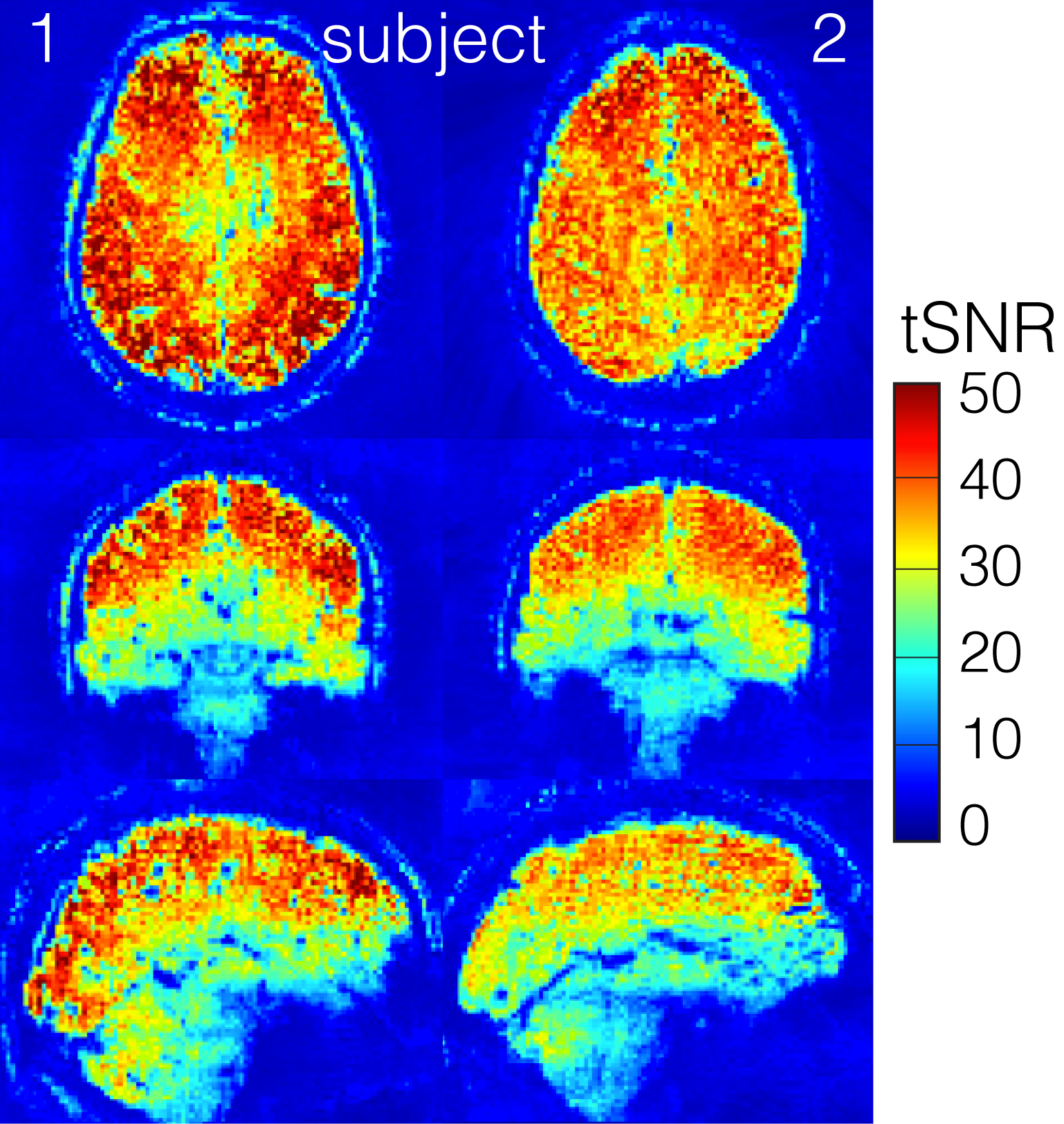
 Supporting Tables:

| Reference | tSNR | Resolution, B_0_ | Other parameters |
| --- | --- | --- | --- |
| *Poser at al. (2010) (1) | 32.4 | 2x2x2 mm^3^, 7T | TR_vol_ = 1.88 s  AF = 2x2 |
| *Van der Zwaag et al. (2012) (4) | 20-30 | 2x2x2 mm^3^, 7T | TR_vol_ = 1.2-4.8 s  AF = 1 |
| *Lutti et al. (2013) (2) | 31 | 1.5x1.5x1.5 mm^3^, 3T | TR_vol_ = 3.2 s  AF = 2 |
| *Jorge et al (2013) (3) | 30-49 | 2x2x2 mm^3^, 7T | TR_vol_ = 3.2 s  AF = 1 |
| Narsude et al. (2016) (48) | 18-31 | 2x2x2 mm^3^, 7T | TR_vol_ = 0.37-3.2 s  AF = 1-6, PF = 1-6/8 |

**Sup. Table S1:** Example 3D EPI tSNR values (for grey matter ROIs) reported in the literature. The studies marked with an asterisk * also report tSNR for 2D EPI protocols, which are included in Sup. Table S2. When comparing values, differences in parameters such as field strength and voxel volume, as well as their impact on physiological noise need to be considered. The last column contains the volume TR as well as the parallel imaging acceleration factor (AF) and amount of partial Fourier (PF) if applicable. When the study used physiological noise correction (Lutti (2), Jorge (3) and Narsude(48)), the tSNR values using physiological noise correction were included. It should be noted that many of these protocols focused on tSNR efficiency (i.e. tSNR per unit time) rather than raw tSNR.

| Reference | tSNR | Resolution, B_0_ | Other parameters |
| --- | --- | --- | --- |
| Krueger at al. (2001) (49) | 84 | 3.4x3.4x4 mm^3^, 3T | TR = 3.0 s  AF = 1 |
| Triantafyllou et al. (2005) (46) | 55 | 2x2x3 mm^3^, 3T | TR = 5.4 s  AF = 1, 6/8 PF |
| *Poser at al. (2010) (1) | 42.5 | 2x2x2 mm^3^, 7T | TR = 3.65 s  AF = 2 |
| Triantafyllou et al. (2011) (47) | 40-75 | 2x2x2 mm^3^, 3T | TR = 2 s  AF = 1-4, 6/8 PF |
| *Van der Zwaag et al. (2012) (4) | 25 | 2x2x2 mm^3^, 7T | TR = 0.15 s  AF = 1 |
| *Lutti et al. (2013) (2) | 24 | 1.5x1.5x1.5 mm^3^, 3T | TR = 3.2 s  AF = 2 |
| *Jorge et al (2013) (3) | 33-44 | 2x2x2 mm^3^, 7T | TR = 3.2 s  AF = 2 |

**Sup. Table S2:** Example 2D EPI tSNR values (for grey matter ROIs) reported in the literature. The studies indicated with an asterisk * also report tSNR for 3D EPI protocols, which are included in Sup. Table S1. See also the caption of Sup. Table S1 for further explanation.
